# Heart Rate Variability in Shift Workers: Responses to Orthostatism and Relationships with Anthropometry, Body Composition, and Blood Pressure

**DOI:** 10.1155/2015/329057

**Published:** 2015-10-01

**Authors:** Nayara Mussi Monteze, Breno Bernardes Souza, Henrique José de Paula Alves, Fernando Luiz Pereira de Oliveira, José Magalhães de Oliveira, Silvia Nascimento de Freitas, Raimundo Marques do Nascimento Neto, Maria Lilian Sales, Gabriela Guerra Leal Souza

**Affiliations:** ^1^School of Nutrition, Federal University of Ouro Preto, 35400-000 Ouro Preto, MG, Brazil; ^2^School of Medicine, Federal University of Ouro Preto, 35400-000 Ouro Preto, MG, Brazil; ^3^Department of Statistics, Federal University of Ouro Preto, 35400-000 Ouro Preto, MG, Brazil; ^4^Institute of Psychiatry, Federal University of Rio de Janeiro, 22290-140 Rio de Janeiro, RJ, Brazil; ^5^Institute of Cancer of São Paulo, 01255-000 São Paulo, SP, Brazil; ^6^Department of Biological Sciences, Federal University of Ouro Preto, 35400-000 Ouro Preto, MG, Brazil

## Abstract

In order to investigate the response of heart rate variability (HRV) components to postural change and their association with cardiovascular risk factors in shift workers, a cross-sectional study with 438 Brazilian males rotating shift workers was done. Anthropometric, body composition, and clinical measures were collected. Electrocardiogram was recorded for 3 minutes, in the supine and orthostatic position, and HRV components were extracted. Descriptive analyses showed that mean values of body mass index, waist circumference (WC), waist-to-height ratio, visceral fat area (VFA), and blood pressure (BP) were higher than the reference values. In the regression model, age, WC, VFA, and systolic BP showed negative association with HRV components. These findings suggest the need for determining effective strategies for the evaluation and promotion of health among shift workers focused on the altered variables.

## 1. Introduction

Heart rate variability (HRV) is a standard noninvasive method that assesses the action of the autonomic nervous system (ANS) on the heart based on variations in the RR interval between consecutive heartbeats [1]. Analysis of HRV on electrocardiogram allows for the decomposition of elements that assess the ANS function on the heart period [2]. A reduction in HRV, and more particularly in vagal modulation, could be considered an indicator of an imbalance in autonomic function and could be associated with an increased risk of morbidity and mortality independent of the presence of well-established risk factors, such as obesity, smoking, and sedentary lifestyle [3–5].

The continuous interplay of sympathetic and parasympathetic activity is crucial to increase or reduce heart performance under different circumstances. Under general physiological conditions, parasympathetic activity is more intense in the resting state and during repairing functions, and sympathetic activity is more intense in situations requiring the mobilization of energy [4]. For instance, during postural change (from supine to orthostatic position), blood pooling occurs in lower parts of the body, which is a normal stimulus that triggers an increase in sympathetic activity in the heart and the vessels, with a consequent rise in blood pressure [6]. Such behavior has been found in some studies, which showed that sympathetic activity was greater in the orthostatic position (90°) and parasympathetic activity prevailed in the supine position (0°) [7, 8]. According to an important review, HRV at rest (wake-sleep and activity-rest) is reduced in shift workers compared to nonshift workers [9]; however, little is known about the causes of these alterations in the shift workers and besides that there is not any study investigating the cardiac autonomic response to postural change in this sample.

Obesity tends to be a strong risk factor for cardiovascular morbidity and mortality in young, middle-aged, and older persons. It has been suggested that ANS dysfunction is an important mediator in the development of obesity associated disease and insulin resistance, although the nature of the link between adiposity and insulin sensitivity is still unclear [10]. Shift work has been associated with deregulation of the circadian rhythm, which could change psychological functions, dietary and social habits, and ANS function and thus contribute to the high risk of cardiovascular diseases [11]. Therefore, it is relevant to investigate the relationship between the HRV components and cardiovascular risk factors (age, obesity, and hypertension) in shift workers.

Therefore, the aims of the present study were to investigate the response of HRV components to postural change and the association of HRV components with age, blood pressure, and obesity indices (anthropometric and body composition variables) in Brazilian shift workers. The hypotheses underlying the present study were as follows: (1) shift workers exhibit deviations in obesity indices, blood pressure, and HRV components relative to reference values; (2) shift work affects the baroreflex mediated response to postural change (transition of supine position to orthostatic position); and (3) HRV components exhibit negative associations with obesity indices, blood pressure, and age. This study has some relevant points: the sample homogeneity (Brazilian, male, rotating shift, and mine worker) and multiple variables collected on the same sample (HRV components, obesity indices, and blood pressure).

## 2. Materials and Methods

### 2.1. Study Design and Sample

This cross-sectional study was conducted with 438 adult Brazilian males who were older than 18 years and worked in shifts. Working in shifts, according to the International Labour Office (ILO) [12], is defined as a “method of organization of working time in which workers succeed one another at the workplace so that the establishment can operate longer than the hours of work individual workers” and can be classified into fixed shift (working time can be organized in two or three fixed shifts: the early, late, and night shifts) or rotating shift (workers might be assigned to work shifts that vary regularly over time: from a shift in the morning, to one in the afternoon, to one at night) [12].

The participants of this study performed rotational shift work as operators of iron ore extraction modern machines without noise exposure. The work regimen included a six-hour shift followed by 12-hour rest. The work shifts and rest periods were rotated, and after each fourth shift the rest period was longer, 36 hours, after which a new cycle began. The full work cycle included four consecutive shifts, in this sequence: 7:00 pm to 1:00 am, 1:00 pm to 7:00 pm, 7:00 am to 1:00 pm, and 1:00 am to 7:00 am.

The study was conducted in the morning of the day with the longest rest period from September 2011 to March 2012. The study complied with the Declaration of Helsinki and was approved by the ethics committee of the institution. All volunteers signed an informed consent form.

### 2.2. Anthropometric Variables

The volunteers' weight, height, and circumferences were measured by trained professionals with volunteers in the orthostatic position and wearing light clothing. The waist circumference (WC) was measured at the mid-point between the last costal arch and the iliac crest [13]. The hip circumference (HC), which was used to calculate the waist-to-hip ratio (WHR), was measured at the level of the greater trochanter [13]. The neck circumference (NC) was measured immediately below the laryngeal prominence [14]. The WHR and the waist-to-height ratio (WHtR) were calculated by dividing the WC by the hip circumference and the WC by the height, respectively [13]. The body mass index (BMI) was calculated by dividing the body weight by the squared height [13].

### 2.3. Body Composition Variables

The body fat mass in kilograms (BFkg) and percentage (BF%) and the visceral fat area (VFA) were calculated by means of segmental tetrapolar bioelectrical impedance using a body composition analyzer InBody model 720 (Biospace Co. Ltd. Factory, Korea) [15].

### 2.4. Clinical Variables

Blood pressure was assessed using an automated digital sphygmomanometer HEM705CP (Omron, Japan) on the right arm with volunteers in the sitting position and after a five-minute rest period. The measurement was performed three times with one-minute interval between measurements [16]. The three measurements of the systolic (SBP) and diastolic (DBP) blood pressure were computed and their means were calculated.

### 2.5. Heart Rate Variability

One PC-compatible computer controlled data acquisition for electrocardiographic parameters using the WinCardio (Micromed, version 4.8.2.8) software program. Electrocardiographic recordings were collected at a sampling frequency of 1000 Hz. An off-line peak detection algorithm (derivative plus threshold) was used to estimate fiducial R-wave points, after which the series was screened by hand and corrected for artifacts. Successive RR intervals were estimated in milliseconds and were converted to heart rate (HR) in beats per minute.

The HRV components were extracted for 3 min in the supine position and for 3 min in the orthostatic position using time domain analysis and frequency domain analysis. The time domain analysis measured changes in the intervals between successive normal RR intervals over time. The parameter used in this study was the root mean square of successive RR interval differences (RMSSD) that reflects parasympathetic activity [2]. The frequency domain method is a spectral method for analysis of the tachogram that provides basic information about how power (variance) distributes as a function of frequency [2]. The components considered in the RR power spectrum in this study were as follows.

- High frequency (HF), from 0.15 to 0.4 Hz, reflects parasympathetic activity [2].
- Low frequency (LF), from 0.04 to 0.15 Hz, could reflect sympathetic activity or a combination of sympathetic and parasympathetic activity [2]. More recently studies have proposed that LF reflects only the parasympathetic activity [17, 18].
- LF/HF reflects sympathetic and parasympathetic balance [2].

Data processing followed the recommendations of the Task Force of the European Society of Cardiology and the North American Society of Pacing Electrophysiology [2]. MATLAB software (KARDIA) was used to analyze cardiac parameters [19].

### 2.6. Procedure

After the employees arrived at the research laboratory and signed the informed consent form, a medical interview was performed to collect information on the use and dosage of medications. Next, body weight, height, WC, HC, NC, BFkg, BF%, and VFA were measured.

Subsequently, after 5 min of resting, blood pressure was measured three times and finally a six-minute electrocardiogram (ECG) was recorded, with three minutes in the supine position and three minutes in the orthostatic position. Although all 12 leads were recorded, only peripheral (or bipolar limb) lead II was used for ECG processing. The employees had fasted for 12–14 hours.

### 2.7. Statistical Methods

The description of variables was presented as median, percentile, and interquartile ranges according to normality as tested by the Shapiro-Wilk test.

After logarithmic normalization of HRV variables, the Wilcoxon test was used to establish whether there were significant differences between the HRV components (logHR, logRMSSD, logHF, logLF, and logLF/HF) in the supine and orthostatic positions.

Spearman's correlation test was used to investigate the presence of a correlation between each HRV component as assessed in the supine position and obesity indices (anthropometric and body composition variables), blood pressure, and age. These correlations were performed only with HRV components in the supine position because these values are more stable and accurate than those in the orthostatic position [2].

Multiple regression models were fitted to investigate the influence of anthropometric and body composition variables, blood pressure, and age (independent variables) on each HRV component as assessed in the supine position (dependent variables). Analysis of residuals of each model was performed to assess validity of assumptions of normality, homoscedasticity, and independence between observations. The statistics Cook distance and variance inflation factor (VIF) were used to identify outliers and to check for possible multicollinearity.

Our analyses considered the wide range of medications reported as modifiers of HRV [20]. Two different physicians classified the medications used by each shift worker, and, in the event of disagreement between them, a third physician was consulted. Different groups of medicines are known to affect HRV in different ways and so, in order to identify the possibility of drug bias, all the analyses were redone after exclusion of workers who were taking medications that could influence the evaluated data.

The data were analyzed using Statistica 7.0 (StatSoft Inc.) and R Development Core Team (2013) software. The significance level was established as 0.05.

## 3. Results

### 3.1. Sample Characteristics

The present study comprised 438 individuals working in rotational shifts. Their age, anthropometric, body composition, and clinical characteristics are described in Table 1. The median age of the sample was 34 years. The median values of BMI, WC, WHtR, VFA, SBP, and DBP were higher than reference values [13–16].

### 3.2. Response of Heart Rate Variability Components to Postural Change

Comparison of HRV components between the supine and orthostatic positions showed increases in the median HR, LF, and LF/HF and reductions in the median RMSSD and HF in the orthostatic compared to the supine position (Table 2).

### 3.3. Relationship between Heart Rate Variability Components and Age, Anthropometric, Body Composition, and Clinical Variables

HR exhibited a positive correlation with all investigated variables except age. RMSSD and HF, which are related to parasympathetic activity, exhibited negative correlations with age, BMI, WC, NC, WHR, WHtR, BFkg, VFA, SBP, and DBP. LF, which is related to sympathetic and parasympathetic activity or to baroreflex function, exhibited negative correlations with age, BMI, WC, WHR, WHtR, and NC. The LF/HF ratio, which is related to autonomic balance, exhibited positive correlations with age, SBP, and DBP. All correlations are described in Table 3.

The fitted regression models showed that HRV components were influenced by age, WC, VFA, and SBP (Table 4). More specifically, VFA and SBP contributed to the increase of HR. VFA and age contributed to the reduction of logRMSSD. Age and WC explained the reduction of logHF and logLF. Finally, age and SBP explained the increase of logLF/HF.

Ninety-three of the 438 employees were using some type of medication. Of those 93, 27 were using one of the following groups of medicines, which were not identified as HRV modifiers: proton pump inhibitors, corticosteroids, nonsteroidal anti-inflammatory drugs, oral anticoagulants, and/or statins. The other 66 were using at least one of the following groups, which can potentially influence HRV [20]: antihypertensive drugs (thiazide-type diuretic; calcium channel blocker; angiotensin-converting enzyme inhibitor; angiotensin receptor blocker; and beta-blockers), anticholinergics, beta2-agonists, antidepressants, anxiolytics, sedative/hypnotics, insulin, oral hypoglycemic agents, and/or levothyroxine. After excluding these 66 employees, we repeated the statistical analyses (data not shown) and noted that the significant results found for the total 438 employees remained. This allowed us to rule out the possibility of drug bias on the findings reported in this study.

## 4. Discussion

The results of the present study pointed to the presence of cardiovascular risk factors in the sample of Brazilian male rotating shift workers, as shown by anthropometry, body composition, and blood pressure, whose median results were higher than the corresponding normal values. In addition, these alterations were shown to correlate with changes in HRV components in the supine position, indicating increased sympathetic and reduced parasympathetic activity. However, the responses of HRV components to the postural change, a baroreflex mediated response, were adequate.

Obesity and hypertension, in addition to other comorbidities such as dyslipidemia and diabetes, are disorders that are frequently found in shift workers [21–23]. In the present study, median values of BMI, WC, WHtR, and VFA, which are indicators of obesity, and SBP and DBP, which are related to hypertension, were above the reference values [13–15], thus corroborating the reports in the literature. The constant presence of those disorders in shift workers is mainly associated with environmental and behavioral changes, as well as deregulation of biological rhythms and the lifestyle to which they are exposed, and these factors may exert a direct influence on their dietary habits, physical activity, sleep quality, mental health, and time available for social interaction [11, 22, 23].

Our findings showed that HRV components (HF, LF, and LF/HF) were qualitatively lower compared to the mean values in a review study with healthy adults [24]. In general, the HRV components vary within a wide range in male shift workers: HR, 58.2 to 82.8 bpm; LF, 323.7 to 1,212 ms²; RMSSD, 29.9 to 70.2 ms; HF, 181.2 to 558.6 ms²; and LF/HF, 1.6 to 3.4 [21, 23, 25, 26]. Values for LF and HF in the present study were substantially lower than the minimum when compared qualitatively with the values described above. The reason for that discrepancy may be associated with the methodological particularities of the various studies, including shift regimen (permanent or rotating), ECG signal processing (application of Task Force guidelines), and sample characteristics, including age and the presence of cardiovascular risk factors.

Nevertheless, the cardiac autonomic response to postural change was adequate, as the values corresponding to parasympathetic components (RMSSD and HF) were higher in the supine compared to the orthostatic position. Those results corroborate HRV at rest as a marker of parasympathetic control of the heart [4]. An increase in LF and LF/HF in the orthostatic position relative to the supine position is controversial [17]. Cysarz et al. [6] and Porta et al. [8] found that the magnitude of tilt interferes with the behavior of LF and LF and LF/HF increased at a 90° tilt; the same increase occurred in the present study with voluntary postural change. To our knowledge, this is the first study to investigate the postural changes in a sample of shift workers. Probably, because postural change is a basic process of cardiac autonomic control, mediated by the baroreflex response, the postural control continued to function properly despite the fact that HRV components in the supine position were found to be lower than expected for healthy adults [24].

Use of RMSSD and HF as indicators of parasympathetic activity is well established [1, 2]. However, the meaning of LF, and consequently also of LF/HF, is still a subject of debate [6, 17, 18, 27]. According to classic studies, LF represents mainly the cardiac sympathetic modulation [5, 28]. Nowadays, some authors have proposed that LF is related to baroreflex function. According to Goldstein et al. [18], with or without adjustment for HF power or respiration, LF power seems to provide an index not of cardiac sympathetic tone but of baroreflex function once manipulations and drugs that change LF power or LF/HF do so not by affecting cardiac autonomic outflows directly but by affecting modulation of those outflows by baroreflex. Piccirillo et al. [27] showed, in a canine experimental acute myocardial infarction model, that, during congestive heart failure, the reduction of LF power and LF/HF ratio probably reflect diminished sinus node responsiveness to autonomic modulation or an abnormal baroreflex function. In another study of the same authors, they showed that LF and HF power were significantly lower seven weeks after acute canine myocardial infarction than at baseline [29]. Other authors have proposed that LF is predominantly associated with parasympathetic activity based on the findings: increases in HF and LF occur after the use of drugs that specifically enhance the cardiac vagal tone; there is similarity of LF values between individuals with cardiac sympathetic denervation and normal baroreflex sensitivity; some validated measures of sympathetic control of the heart, such as plasma epinephrine levels and cardiac norepinephrine spillover, are not correlated with LF [17, 18]. Therefore, more studies are necessary to clarify the origins and clinical significance of LF.

Among the variables that exhibited a correlation with HRV, the following remained significant following multiple linear regression analyses: age exhibited negative correlations with logRMSSD, logHF, and logLF and a positive correlation with LF/HF; WC exhibited negative correlations with logHF and logLF; VFA exhibited a negative correlation with logRMSSD and a positive correlation with HR; and SBP exhibited positive correlations with HR and LF/HF. These results indicate that, in the present sample, age, WC, VFA, and SBP were the variables with the strongest influence on HRV.

Similar to our study, Kim et al. [26] also observed that greater age is associated with lower HRV, parasympathetic activity, and baroreflex sensitivity in particular.

The findings about the WC and VFA could indicate that higher values of these anthropometric and body composition parameters are associated with less activity of HRV, specially the components that reflect parasympathetic activity. Similar results were reported by Ramos and Araújo [30], who found that cardiac vagal components decrease in parallel with increases in BMI, the sum of skinfold measurements (reflecting total body fat), and WC. Additionally, other studies found a relationship between WC, BF, and WHR and HRV vagal components [5, 26, 31].

With regard to blood pressure, SBP and DBP exhibited positive association with HR and LF/HF. Yue et al. [32] assessed the HRV in hypertensive and nonhypertensive patients and found increased sympathetic and decreased parasympathetic activity in hypertensive patients, compared to normotensive patients. In the same line, Thiyagarajan et al. [33] found that young adults with prehypertension had decreased cardiovagal modulation (HF, RMSSD, and SDNN), increased LF/HF ratio, and elevated cardiovascular risk factors comparable to the normotensive group.

Some limitations must be discussed about our findings. First, as the sample is composed only of male shift workers with the same work arrangement of alternating shifts, comparisons of gender and shift work specificities (permanent or alternating, clockwise or counterclockwise, etc.) could not be performed. Second, although the employees had similar environmental and work conditions (they were all operators of iron ore extraction machinery working in air-conditioned trucks with insulation against sound, water, and dust), data on behavioral factors (e.g., eating habits, physical activity, sleep-wake pattern, and smoking) was not collected. This information could be useful in determining potential mediators of the associations found. Third, we did not measure breathing during the electrocardiographic recordings. Although it is known that breathing (respiratory frequency and volume) potentially influences the HRV variables, Penttilä et al. [34] demonstrated that RMSSD values are not affected by the respiratory pattern. In the present study, both parasympathetic variables collected (HF and RMSSD) showed the same statistical tendencies; therefore, we can infer that the lack of breathing register most likely did not bias the study results.

## 5. Conclusion

Our findings showed that the cardiac autonomic response to postural change in shift workers was adequate. Nevertheless, they exhibited alterations in the assessed anthropometric, body composition, clinical, and HRV variables compared to the reference values. Regression analysis showed that age, obesity (WC and VFA), and SBP account for a significant part of the reduction of HRV components in this population. Even when taking into consideration the full complexity and variety of factors that may be involved in those alterations, the need to provide comprehensive healthcare to shift workers is undeniable, including specific occupational strategies to minimize the impact of these cardiovascular risk factors on their health and ensure good quality of life.

## Figures and Tables

**Table 1:** Sample characteristics.

Variables	Median (p25; p75)	Quartile range
Age (years)	34.00 (31.00, 39.00)	8.00
Anthropometric		
Height (m)	1.74 (1.70, 1.79)	0.09
Body weight (kg)	80.80 (73.60, 89.30)	15.70
Body mass index (kg/m²)	26.70 (24.40, 29.20)	4.80
Waist circumference (cm)	92.00 (85.50, 97.50)	12.00
Hip circumference (cm)	102.50 (98.30, 107.00)	8.75
Neck circumference (cm)	39.25 (37.50, 41.15)	3.65
Waist-to-hip ratio	0.89 (0.86, 0.93)	0.07
Waist-to-height ratio	0.53 (0.49, 0.56)	0.07
Body composition		
Total body fat (%)	23.55 (19.20, 28.20)	9.00
Total body fat (kg)	18.95 (14.60, 24.30)	9.70
Visceral fat area (cm²)	121.25 (95.40, 147.70)	52.30
Clinical		
Systolic blood pressure (mmHg)	131.67 (122.67, 141.33)	18.70
Diastolic blood pressure (mmHg)	82.33 (76.00, 88.33)	12.30

**Table 2:** Medians of heart rate variability (HRV) variables in the supine and orthostatic positions.

HRV variables	Supine position	Orthostatic position	*p* value
	Median (p25, p75)	Median (p25, p75)	
HR (bpm)	65.9 (59.7, 71.9)	82.3 (74.2, 90.2)	<0.0001
RMSSD (ms)	34.3 (23.9, 50.1)	19.9 (13.3, 30.1)	<0.0001
HF (ms²)	81.9 (35.7, 168.7)	26.1 (10.5, 62.1)	<0.0001
LF (ms²)	103.4 (60.8, 186.4)	120.6 (62.4, 224.1)	0.005
LF/HF	1.3 (0.7, 2.5)	4.7 (2.5, 7.9)	<0.0001

HRV: heart rate variability; HR: heart rate; RMSSD: root mean square of successive RR interval differences; HF: high frequency; LF: low frequency; LF/HF: ratio of low frequency and high frequency. *Rawdata.* The Wilcoxon test was made using log transformation values, but, for ease of interpretation, all medians (plus p25, p75) are given in the original units.

**Table 3:** Spearman correlation coefficients (Rho) between heart rate variability measures (supine position) and age, anthropometric, body composition, and clinical variables.

Variables	log⁡HR	log⁡RMSSD	log⁡HF	log⁡LF	log⁡LF/HF
Age	−0.04	−0.27^∗^	−0.24^∗^	−0.17^∗^	0.13^∗^
Anthropometric					
Body mass index (kg/m²)	0.18^∗^	−0.14^∗^	−0.14^∗^	−0.12^∗^	0.05
Waist circumference (cm)	0.22^∗^	−0.17^∗^	−0.16^∗^	−0.15^∗^	0.05
Neck circumference (cm)	0.19^∗^	−0.15^∗^	−0.14^∗^	−0.13^∗^	0.04
Waist-to-hip ratio	0.20^∗^	−0.18^∗^	−0.15^∗^	−0.15^∗^	0.05
Waist-to-height ratio	0.21^∗^	−0.15^∗^	−0.15^∗^	−0.14^∗^	0.05
Body composition					
Total body fat (%)	0.15^∗^	−0.06	−0.07	−0.03	0.03
Total body fat (kg)	0.17^∗^	−0.09^∗^	−0.11^∗^	−0.08	0.03
Visceral fat area (cm²)	0.24^∗^	−0.13^∗^	−0.11^∗^	−0.09	0.04
Clinical					
Systolic blood pressure (mmHg)	0.21^∗^	−0.13^∗^	−0.14^∗^	−0.002	0.16^∗^
Diastolic blood pressure (mmHg)	0.18^∗^	−0.16^∗^	−0.16^∗^	−0.06	0.11^∗^

log⁡HF, natural logarithm of power in the high frequency range; log⁡HR, natural logarithm of the heart rate; log⁡LF, natural logarithm of power in the low frequency range; log⁡LF/HF, natural logarithm of the ratio LF (ms²)/HF (ms²); log⁡RMSSD, natural logarithm of the root mean square of successive RR interval differences; ∗ represents *p* < 0.05.

**Table 4:** Equations using regression analysis for cardiac variables.

Regression models	*β* ± SE	*p* value	*R* ^2^ adjusted
HR			
Constant	47.3 ± 4.172	**<0.001**	8.4%
Visceral fat area (cm²)	0.0459 ± 0.01109	**<0.001**	
Systolic blood pressure (mmHg)	0.101 ± 0.03195	**0.002**	
log⁡RMSSD			
Constant	2.018 ± 0.067	**<0.001**	10.3%
Age (years)	−0.010 ± 0.001	**<0.001**	
Visceral fat area (cm²)	−0.0008 ± 0.0002	**0.002**	
log⁡HF			
Constant	3.098 ± 0.242	**<0.001**	8.0%
Age (years)	−0.018 ± 0.003	**<0.001**	
Waist circumference (cm)	−0.005 ± 0.002	**0.016**	
log⁡LF			
Constant	2.85 ± 0.193	**<0.001**	4.5%
Age (years)	−0.009 ± 0.002	**0.001**	
Waist circumference (cm)	−0.005 ± 0.002	**0.006**	
log⁡LF/HF			
Constant	−0.652 ± 0.181	**<0.001**	4.3%
Age (years)	0.008 ± 0.002	**0.001**	
Systolic blood pressure (mmHg)	0.003 ± 0.001	**0.004**	

log⁡HF: natural logarithm of power in the high frequency range; log⁡HR, natural logarithm of the heart rate; log⁡LF: natural logarithm of power in the low frequency range; log⁡LF/HF: natural logarithm of the ratio LF (ms²)/HF (ms²); log⁡RMSSD: natural logarithm of the root mean square of successive RR interval differences. Numbers in bold represent *p* < 0.05.
